# Assurance calculations for planning clinical trials with time-to-event outcomes

**DOI:** 10.1002/sim.5916

**Published:** 2013-07-16

**Authors:** Shijie Ren, Jeremy E Oakley

**Affiliations:** a School of Health and Related Research, University of SheffieldRegent Court, 30 Regent Street, Sheffield, S1 4DA, U.K.; b School of Mathematics and Statistics, University of SheffieldThe Hicks Building, Hounsfield Road, Sheffield, S3 7RH, U.K.

**Keywords:** assurance, elicitation, prior distribution, power, sample size, survival analysis

## Abstract

We consider the use of the assurance method in clinical trial planning. In the assurance method, which is an alternative to a power calculation, we calculate the probability of a clinical trial resulting in a successful outcome, via eliciting a prior probability distribution about the relevant treatment effect. This is typically a hybrid Bayesian-frequentist procedure, in that it is usually assumed that the trial data will be analysed using a frequentist hypothesis test, so that the prior distribution is only used to calculate the probability of observing the desired outcome in the frequentist test. We argue that assessing the probability of a successful clinical trial is a useful part of the trial planning process. We develop assurance methods to accommodate survival outcome measures, assuming both parametric and nonparametric models. We also develop prior elicitation procedures for each survival model so that the assurance calculations can be performed more easily and reliably. We have made free software available for implementing our methods.

## 1. Introduction

Sample size determination is an important part of clinical trial design and conventionally involves power calculations. However, the power of a trial does not necessarily give the probability of the trial demonstrating a treatment effect, as the true treatment effect may be different to that assumed in the power calculation. Several authors have proposed a hybrid classical-Bayesian approach for assessing the probability of a successful trial, given the sample size only, which can then be used to inform sample size decisions.

The hybrid method was first considered by Spiegelhalter and Freedman 1. They constructed an unconditional probability of having a desired outcome and called this unconditional probability the average power. O’Hagan and Stevens 2 used this method for choosing sample sizes for clinical trials of cost-effectiveness. They referred to the unconditional probability of a successful trial as the ‘assurance’ of the trial, and we use this term here. O’Hagan *et al.* 3 extended assurance methods to two-sided testing and equivalence trials, covering the use of non-conjugate prior distributions for uncertain parameters. Chuang-Stein 4 discussed the difference between traditional power calculations and assurance calculations to determine sample sizes, giving an example of planning the next trial based on the results of an early trial. Chuang-Stein and Yang 5 reviewed the concept of assurance and illustrated its use when planning phase III trials. They also applied assurance to study designs when re-estimating a sample size based on an interim analysis.

An assurance calculation requires a prior distribution for the treatment effect, but does not necessarily involve a Bayesian analysis of the trial data. The method of analysis, and in particular the criteria for which the trial is deemed a ‘success’, are determined externally, for example, by a regulator. Once the criteria have been specified, a prior distribution is used to assess the probability that these criteria will be met. Typically, the prior distribution will only be used in the design stage and not the analysis. At the design stage, the risk of trial failure is primarily the trial sponsor’s, and so it should be uncontroversial for a trial sponsor to use all their prior knowledge in assessing such a risk.

We consider clinical trials in which the endpoint of interest is a survival time. For time-to-event outcome measures, power and sample size calculations have been well studied under various model assumptions. For example, Schoenfeld and Richter 6 developed a power function with a limited recruitment period and a pre-specified follow-up period under the assumption that the survival times in each treatment group follow exponential distributions and patients enter the trial uniformly. Gross and Clark 7 provided a method of calculating sample size by assuming that the sample mean survival time is approximately normally distributed under Weibull models for the survival times. Freedman 8 and Schoenfeld 9 derived sample size formulae under the assumption of proportional hazards based on asymptotic properties of the logrank statistic.

Little has been done in calculating assurance for survival endpoints. Assuming proportional hazards, Spiegelhalter *et al.* 10 derived an assurance formula in the case of equal allocation and follow-up. The only uncertain variable considered was the log hazard ratio, and a normal prior was assumed. In this paper, we extend assurance calculations to accommodate both parametric and proportional hazards models. Under proportional hazards models, we derive an assurance formula assuming uniform patient entry over a limited recruitment period. We consider uncertainty in both the log hazard ratio and the baseline survivor function.

In Section 2, we review how assurance is calculated to determine the unconditional probability of having a desired outcome. In Section 3, we derive assurance calculations for exponential and Weibull survival models and describe the elicitation methods for the required prior distributions. In Section 4, we extend assurance calculations to accommodate proportional hazards models, considering uncertainty in both treatment effect and baseline survivor function. We also describe the procedure of generating the baseline survivor function. Examples are given in Section 5.

## 2. Assurance and sample size

We now review the concept of assurance. Suppose that a randomised controlled trial is to be conducted to compare an experimental treatment and a standard treatment for a particular disease. A hypothesis test is to be carried out to test the null hypothesis that the treatment effect *θ* = 0 against the alternative that *θ* ≠ 0. On the basis of a power calculation, the sample size is chosen to solve

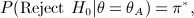
(1) for some desired probability π*.

The power of the test *P*(Reject *H*_0_ | *θ* = *θ*_*A*_) provides the probability of successfully rejecting the null hypothesis if the true value of *θ* is the specified *θ*_*A*_. As the true value of *θ* may be very different to *θ*_*A*_, the actually probability of successfully rejecting the null hypothesis may be very different to the power.

Assurance is the unconditional probability that the trial will end with the desired outcome, which we derive via

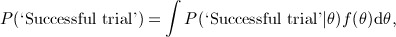
(2) where *f*(*θ*) is the prior distribution for the true treatment effect *θ*. If a successful trial simply corresponds to rejecting a null hypothesis of no treatment effect, then the assurance in ) can be thought of as an expected power (interpreting *θ*_*A*_ in as the true value of the treatment effect, rather than some minimum clinically relevant difference).

If our desired outcome is to reject the null hypothesis with data favouring the experimental treatment, then assurance is given by

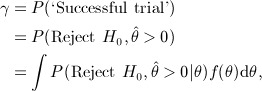
 with 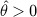 indicating that the data favour the experimental treatment. We again emphasise that specifying what constitutes a ‘successful trial’ is not part of the assurance method; the criteria determining a successful trial are set externally, and the idea of assurance is to use prior information to determine the probability that these criteria are met.

The power of a clinical trial can, in theory, be made as large as desired by increasing the sample size. The same does not hold for assurance. For a large enough sample size, we will ‘observe’ the true treatment effect, so that the assurance converges to the prior probability that the new treatment is suitably effective. If this prior probability is low, no trial will have a high assurance of success; we cannot ‘beat the prior’.

We restrict the scope of this paper to frequentist methods for analysing the trial data, but Bayesian methods could be used and are discussed in depth by Ibrahim *et al.* 11 and Christensen *et al.* 12. (In particular, Ibrahim *et al.* 11 discussed Bayesian inference for all the survival models considered in this paper.) In this case, there may be a distinction between the prior used in the design stage, and the prior used in the analysis stage, if the regulator is not willing to accept the trial sponsor’s prior.

## 3. Assurance calculations for parametric survival models

We now suppose that, in each of two treatment groups, the outcome variable for each patient is the survival time to some event and consider exponential and Weibull models for the survival times. For each model, we first choose the analysis method, and hence the criteria for a successful trial. We then consider assurance calculations and elicitation methods for the required prior distributions.

### 3.1 Exponential distribution

We first suppose that the survival times in each treatment group follow an exponential distribution, with hazard rates *λ*_1_ and *λ*_2_ ( *i* = 1 for the control group and *i* = 2 for the experimental group) and allow for a limited recruitment period from time 0 to *R* with uniform patient entry and *T* as the total trial length. The time origin for survival time is when a patient enters the trial, not when the trial starts. Here, we consider the analysis method based on Schoenfeld and Richter 6.

The null hypothesis is *m*_1_ / *m*_2_ = 1, where *m*_*i*_ is the median survival time in group *i*, against the alternative *m*_1_ / *m*_2_ = *ϕ*, where *ϕ* is the minimum clinically important difference. Note that assuming an exponential model, the hypotheses stated earlier are equivalent to *H*_0_ : *θ* = 0 versus *H*_1_ : *θ* ≠ 0, where

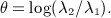
(3) The test statistic is

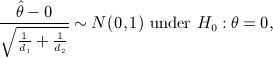

where *d*_*i*_ is the number of events in group *i* and 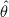 is the maximum likelihood estimate of *θ*.

On the basis of the asymptotic properties of the test statistic, the power *π*^*E*^ of a 100*α%* two-sided test is

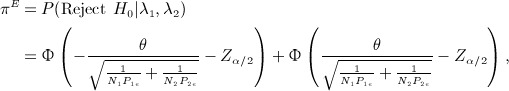
(4) where *N*_*i*_ is the number of patients in group *i* and *P*_*ie*_ is the probability of an individual patient in group *i* experiencing the outcome event during the trial. Schoenfeld and Richter 6 derived an exact formula for *P*_*ie*_:

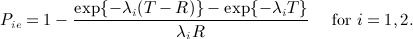
(5)

The assurance of rejecting the null hypothesis with data favouring the experimental treatment is

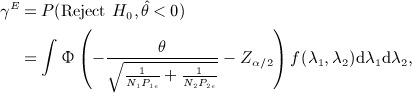
(6) where *θ* and *P*_*ie*_ are functions of *λ*_1_ and *λ*_2_.

#### 3.1.1 Constructing the priors

From, we see that a joint prior is required for *λ*_1_ and *λ*_2_. Kadane and Wolfson 13 argued that it is better to ask for opinion about observable quantities rather than parameters in statistical models, and we follow their advice here. We elicit *f*(*λ*_1_,*λ*_2_) via judgements about survival rates at some specified time.

To construct a prior for each parameter, first note that

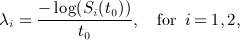
(7) with *S*_*i*_(*t*_0_) the survival rate for group *i* at time *t*_0_. Hence, to elicit the joint prior distribution, we first elicit judgements about *S*_1_(*t*_0_) instead of eliciting beliefs about *λ*_1_ directly. An expert may judge *S*_1_(*t*_0_) to be informative for *S*_2_(*t*_0_), so that *λ*_1_ and *λ*_2_ are not independent. To elicit this dependence, we propose to elicit judgements about the difference, *ρ* = *S*_2_(*t*_0_) − *S*_1_(*t*_0_), and assume that *ρ* is independent of *S*_1_(*t*_0_).

Methods for eliciting univariate distributions are given in Sections 6.3 and 6.4 of O’Hagan *et al.* 14 and can be implemented using the freely available SHELF package 15 and the MATCH online elicitation tool available at http://optics.eee.nottingham.ac.uk/match/uncertainty.php. See also Johnson *et al.* 16 for a systematic review of elicitation methods.

One option is to elicit a beta distribution for *S*_1_(*t*_0_) and a normal distribution for *ρ*, truncating the normal prior if necessary to ensure *S*_2_(*t*_0_) ∈ (0,1). (An alternative would be to use a shifted and scaled beta distribution for *ρ*, although we have not found the need to truncate to cause significant computational problems.)

For illustration, we describe a ‘trial roulette’ method proposed by Gore 17 to elicit a normal prior for *ρ*. The method is based on the fixed interval approach, in which the expert is asked to provide a probability that the unknown quantity of interest will fall in a pre-fixed interval. Using the SHELF package, the facilitator, who conducts the elicitation, firstly elicits from the expert the lower and upper bounds of the range of plausible values for *ρ*. Then the facilitator divides the range from the lower bound to upper bound in to 10 equal-width ‘bins’. The expert is asked to specify his or her probability of *ρ* lying in a particular bin by placing ‘chips’ in that bin, with the proportion of chips allocated representing the probability. The number of chips given to the expert is specified by the facilitator. For example, if in total 20 chips are used, then each chip represents a probability of 0.05. The trial roulette method is simple to use and provides the expert with an immediate display of her elicited judgements.

A parametric distributed can be fitted to the elicited judgements using a least squares procedure: the parameters are chosen to make the fitted probabilities as close as possible to the elicited probabilities. Feedback should be provided to the expert for checking the adequacy of the elicited distribution.

One illustration of the use of elicitation in clinical trials is given by Parmar *et al.* 18, who used a questionnaire to elicit the log hazard ratio given in by eliciting a point estimate for *S*_1_(*t*_0_) and a prior distribution for *ρ* using the roulette method. Tan *et al.* 19 and Hiance *et al.* 20 both adapted this questionnaire and used it for a phase III trial. Our elicitation process is more complicated, as we consider uncertainty in both *S*_1_(*t*_0_) and *ρ*.

Given the elicited distributions *f*(*S*_1_(*t*_0_)) and *f*(*ρ*), we estimate *γ*^*E*^ using Monte Carlo simulation:

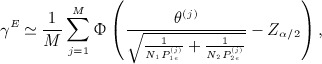
(8) where *θ*^(*j*)^ and 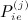 for *i* = 1,2, are obtained by the following steps.

1. Simulate 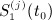 from the elicited prior distribution *f*(*S*_1_(*t*_0_)).
2. Simulate *ρ*^(*j*)^ from the elicited prior distribution *f*(*ρ*) and calculate 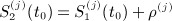.
3. Calculate 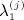 and 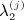 using.
4. Calculate *θ*^(*j*)^ using.
5. Calculate 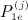 and 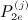 using (5).

The process is computationally quick, so we can make *M* very large to ensure convergence.

### 3.2. Supporting software

We have made available software to implement the methods in this paper. The software can be downloaded from www.jeremy-oakley.staff.shef.ac.uk/assurance.zip. For the exponential case, we have a written an interactive elicitation tool for computing assurance. The code is written in R 21 and uses the rpanel package of Bowman and Crawford 22 and the tkrplot package of Tierney 23 to provide interactive graphics. The tool helps to elicit the prior distributions of the baseline survival rate *S*_1_(*t*_0_) at a specified time *t*_0_, and the survival difference *ρ* between the experimental group and the control group at time *t*_0_, using the trial roulette method. The tool also provides feedback to check the adequacy of the elicited distributions. Once the priors are specified, the tool draws both power and assurance curves for the corresponding elicited distributions. Users can see immediately how changes in the elicited beliefs affect the assurance.

### 3.3. Weibull distribution

We now suppose that the survival times of patients receiving the standard and experimental treatment follow Weibull distributions, with scale parameters *λ*_1_, *λ*_2_ and shape parameters *κ*_1_, *κ*_2_, respectively. The probability density function of the Weibull distribution in each treatment group is

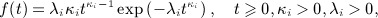
 for *i* = 1,2. The method of analysis that we consider here is to compare mean survival times for each group. We assume that the sample mean survival times are approximately normally distributed: 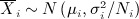, with *N*_*i*_ the number of patients in group *i* ( *i* = 1 for the control group, *i* = 2 for the experimental group).

Gross and Clark 7 derived a power function of a 100*α%* two-sided test of the null hypothesis that the mean survival times are the same, *μ*_1_ = *μ*_2_, against the alternative *μ*_1_ ≠ *μ*_2_. They used the test statistic

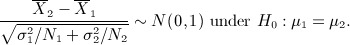


The power formula is

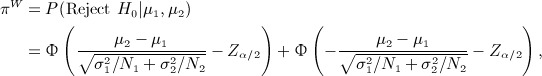
(9) where *μ*_*i*_ and 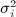, for *i* = 1,2, are expressed by

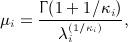
(10)

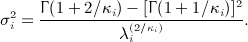
(11)

As the variance parameters 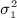 and 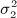 are unknown, we switch to using a two-sample *t*-test in the assurance calculation. The assurance of rejecting the null hypothesis with data favouring the experimental treatment is given by

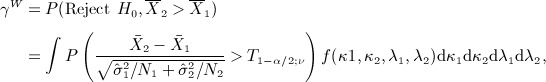
(12) where *T*_1 − *α* / 2;*ν*_ is the 100 × (1 − *α* / 2) percentile from the *t*-distribution with degrees of freedom *ν* calculated according to Welch’s *t*-test. As in the exponential case, we estimate this integral using Monte Carlo simulation.

#### 3.3.1. Constructing the priors

To derive the assurance, a joint prior distribution for *κ*_1_,*κ*_2_,*λ*_1_ and *λ*_2_ is needed. Clearly, making judgements directly about these parameters would be too difficult, so we again construct the priors from judgments about survival rates.

Several authors have presented methods for eliciting an expert’s opinion for a single Weibull distribution. In Singpurwalla 24, beliefs about the median survival time and shape parameter *κ* are elicited. Berger and Sun 25 and Kaminskiy and Krivtsov 26 both considered a predictive approach, in which survival rates at two specified times are elicited. We consider a similar approach, allowing for the possibility of dependence between the two uncertain survival distributions.

For each group, the shape and scale parameters can be estimated using the survival rate after two periods. Let *S*_*i*_(*t*_0_) and *S*_*i*_(*t* ′ _0_) be the survival rates at time *t*_0_ and *t* ′ _0_, where *t* ′ _0_ > *t*_0_ without loss of generality. The Weibull parameters are derived from

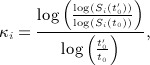
(13)

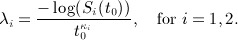
(14)

We could elicit the prior distribution for *κ*_*i*_ and *λ*_*i*_ by eliciting an expert’s opinion about *S*_*i*_(*t*_0_) and *S*_*i*_(*t* ′ _0_) and then applying and, but the expert may judge that *S*_*i*_(*t*_0_) and *S*_*i*_(*t* ′ _0_) are dependent. Instead, we suggest eliciting beliefs about the following four observable quantities (assuming independence):

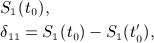
(15)

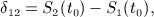
(16)

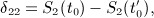
(17) and this will induce a joint prior for *κ*_1_, *κ*_2_, *λ*_1_ and *λ*_2_. Another option is to elicit judgments about odds ratios instead of differences.

If beliefs about the differences between two survival rates are elicited, one option is to elicit a beta distribution for *S*_1_(*t*_0_), and a normal prior for *δ*_12_, beta priors for *δ*_11_, *δ*_22_. It may be necessary to truncate the priors to ensure *S*_1_(*t* ′ _0_), *S*_2_(*t*_0_) and *S*_2_(*t* ′ _0_) all in the range (0,1), but this is unlikely to be a significant computational problem. If the odds ratios are the uncertain quantities of interests, we could again elicit a beta distribution for *S*_1_(*t*_0_), and lognormal distributions for the odds ratios.

We estimate *γ*^*W*^ using Monte Carlo simulation:

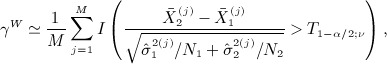
(18) where *I*() is the indicator function, and the simulation procedure for each *j* is as follows.

1. Simulate 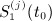, 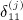, 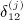 and 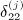 from their elicited prior distribution.
2. Calculate 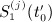, 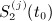 and 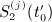 from the sampled values in step 1 using Equations (15)–(17).
3. Calculate 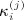 and 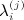 for *i* = 1,2, using Equations (13) and (14).
4. Simulate survival times 
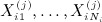
 for *i* = 1,2, for the two groups, from Weibull distributions with the parameter values calculated in step 3.
5. Calculate the sample means 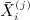 and sample variances 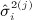 for *i* = 1,2, from the simulated data in step 4.

Again, the process is computationally quick, so *M* can be chosen to be very large to ensure convergence. The R code to compute the assurance for the Weibull model is also available in the supporting software described in Section 3.2.

### 3.4. Priors based on historical data

Suitable historical data may be available for informing the prior distributions, particularly with regard to the control arm of a trial. We could then derive a posterior distribution given the historical data (following the approaches described by Ibrahim *et al.* 11), which could be used as the prior in the assurance calculation. For example, in the exponential case, the distribution for the rate parameter *λ*_1_ in the control arm could be based on the historical data (and perhaps a noninformative prior), and we would then only elicit prior judgements about the difference between the treatments *ρ* = *S*_2_(*t*_0_) − *S*_1_(*t*_0_).

Ibrahim *et al.* 11) also described the use of ‘power priors’. This also involves deriving a posterior distribution given the historical data, but a posterior in which the likelihood function is downweighted, by raising it to a power between 0 and 1. The downweighting may be used to reflect differences between the study populations in the historical and new trials.

## 4. Assurance calculation for proportional hazard and nonparametric survivor function models

We now consider a proportional hazards model, with no parametric assumption about the underlying survivor functions, for a two-arm trial with uniform patient entry during the recruitment period 0 to *R* and total study length *T*. We suppose that the trial results will be analysed with a logrank test. Let *h*_*i*_ and *S*_*i*_ denote the hazard and survivor function for treatment group *i* ( *i* = 1 for the control group and *i* = 2 for the experimental group), respectively, with *ϕ* the hazard ratio *h*_2_(*t*) / *h*_1_(*t*). A two-sided 100*α%* logrank test is performed to test the null hypothesis, *H*_0_, that the log hazard ratio, *θ* = log(*ϕ*), is zero against the alternative *θ* ≠ 0.

Assuming equal number of patients per treatment group, the power formula is

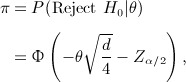
 where *d* is the total number of events in the trial. Spiegelhalter *et al.* 10 derived an exact assurance formula assuming a normal prior *N*(*m*,*v*) for *θ*:

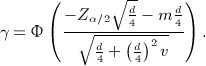
 This assumes that all patients are monitored until the outcome event with no limitation of the length of the trial. We extend this formula to allow for limited recruitment and follow-up periods.

Our assurance calculation is based on the power formula derived by Schoenfeld 9. The recruitment rate is uniform over an interval 0 to *R*, and there is a follow-up period to time *T*. The time origin for survival time is when a patient enters the trial. Given the total number of patients *N*, the test statistic is

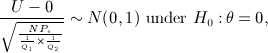

where *U* is the logrank statistic, *P*_*e*_ is the probability that an individual patient will experience the outcome event during the trial and *Q*_*i*_ is the proportion of patients allocated to group *i* for *i* = 1,2. Let *P*_1*e*_ and *P*_2*e*_ denote the probabilities that a patient from treatment groups 1 and 2 will experience the outcome event during the trial, respectively. The power formula for a two-sided 100*α%* logrank test is

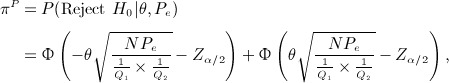
(19) where

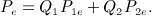
(20)

The assurance, considering uncertainty in *θ* and *P*_*e*_, is

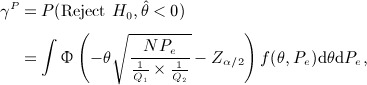
(21) where 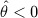 implies that the experimental treatment is better than the standard/placebo.

To compute the assurance *γ*^*P*^, a joint prior for *θ* and *P*_*e*_ is required. Depending on whether there are data available, the elicitation procedures for *P*_*e*_ are different. When there are no data available about the standard treatment, we could elicit *P*_*e*_ directly, and the assurance is calculated using Equation (28). When data about the standard treatment are available, we will use the data to learn about *S*_1_(.) and then derive *P*_*e*_ from

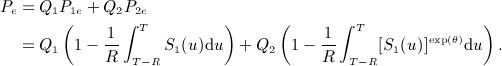
(22)

With both the data available and no data available cases, beliefs about the log hazard ratio *θ* are required. The model parameter *θ* can be expressed in terms of the survival rates at a fixed time point *t*_0_ in each group:

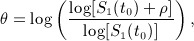
(23) where *ρ* denotes the difference between the survival rates in the two groups at time *t*_0_ (group 2 minus group 1). We elicit opinion about the survival rates *S*_1_(*t*_0_) and *ρ* instead of the model parameter *θ* as in the case of exponential models.

### 4.1. Constructing the priors with no data available

From (28), a joint prior distribution for *θ* and *P*_*e*_ is required. Under the proportional hazards model, *P*_2*e*_ is given by

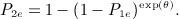
(24) Hence, to elicit the joint prior distribution for *θ* and *P*_*e*_, we can elicit beliefs about *P*_1*e*_, *S*_1_(*t*_0_) and *ρ* and then apply (31), (30) and . As in Section 3.1.1, we could elicit independent beta distributions for *P*_1*e*_ and *S*_1_(*t*_0_) and an independent normal distribution for *ρ*. As before, a Monte Carlo simulation can be used to estimate (21).

### 4.2. Constructing the priors with available data

Information from a pilot study or historic data for the standard treatment may be available at the planning stage. In this section, we describe a method of incorporating both information from the data and expert opinion to obtain the final joint prior distribution for the assurance calculation.

From (22), *P*_*e*_ is determined by *S*_1_(.) and *θ*. Hence, we consider a joint distribution for (*θ*,*S*_1_(.)) rather than (*θ*,*P*_*e*_). The integral in the first term of (22) can be estimated numerically, for example, using Simpson’s rule:

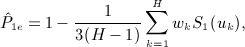
(25) where *w*_*k*_ = 1,4,2,4,2 …,4,1 for *k* = 1, …,*H*, and *H* is the number of subintervals in the interval (*T* − *R*) to *T* with *H* an odd number. We now just require a joint prior for (*θ*,*S*_1_(*u*_1_), …,*S*_1_(*u*_*H*_)).

Taking into account both uncertainty in the log hazard ratio *θ* and baseline survival rates *S*_1_(*u*_*k*_) for *k* = 1, …,*H*, the (approximate) assurance 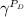 is

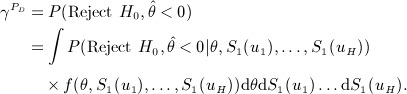
(26)

We suppose that survival data (with right censoring at *J* distinct times, *τ*_1_, …,*τ*_*J*_) are available for the standard treatment, and so consider inference for *S*_1_(*u*_1_), …,*S*_1_(*u*_*H*_) using a Dirichlet distribution, as in Susarla and Ryzin 27. To handle censoring, we use a Gibbs sampling approach, as suggested by Kuo and Smith 28, to generate (*S*_1_(*u*_1_), …,*S*_1_(*u*_*H*_)) from its posterior distribution, which is another Dirichlet distribution. See also Ibrahim *et al.* 11 for further discussion of inference for survivor functions using Dirichlet distributions.

The problem with censored data is that we do not observe the exact event times. Hence, in the Gibbs sampler, we firstly simulate event times for each censored observation and then update the prior distribution using these simulated times and the observed uncensored event times.

We now describe the procedure for simulating (*S*_1_(*u*_1_), …,*S*_1_(*u*_*H*_)) using the Gibbs sampling approach. The first step is to partition the sample space [(*T* − *R*),*T*] into (*H* + *J*) subintervals according to the censored times and the quadrature points in Simpson’s rule. The uncertain quantities of interest are *S*_1_(*u*_1_), …,*S*_1_(*u*_*H*_), and the nuisance parameters are *S*_1_(*τ*_1_), …,*S*_1_(*τ*_*J*_). To simplify the notation, we define

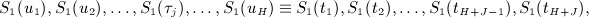

where *t*_*j*_ is the *j*th smallest value in the set {*u*_1_, …,*u*_*H*_,*τ*_1_, …,*τ*_*J*_}.

We define a vector of probabilities *p*_1:*H* + *J* + 1_ = (*p*_1_,*p*_2_, …,*p*_*H* + *J* + 1_) of an event occurring in each subinterval:

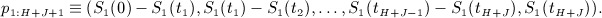


We consider a Dirichlet process prior for *S*_1_(.) with parameter function *α*, of the form *α*([*t*, ∞ )) = *c*_0_*G*(*t*). The function *G*(.) represents the beliefs about the shape of *S*_1_(.). The precision parameter *c*_0_ is a positive real number, and it measures how much weight to put on these prior beliefs. The prior distribution of *p*_1:*H* + *J* + 1_ is a Dirichlet distribution:

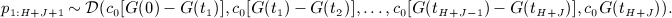


In the Gibbs sampler, we iterate between sampling event times for the censored data conditional on the probabilities *p*_1:*H* + *J* + 1_, and sampling a new probability vector *p*_1:*H* + *J* + 1_ given the sampled event times.

#### 4.2.1. Sampling the unobserved event times for the censored data

For the Gibbs sampler, conditional on the probabilities *p*_1:*H* + *J* + 1_, we need to sample which interval each censored event time occurred in (we do not actually need the precise event time). We introduce variables *Z*_*k* + 1,*k*_, …,*Z*_*H* + *J* + 1,*k*_, which decompose the number of censored observations *r*_*k*_ in the interval (*t*_*k* − 1_,*t*_*k*_], into the number of events that fall in the intervals (*t*_*k*_,*t*_*k* + 1_], …, (*t*_*H* + *J* − 1_,*t*_*H* + *J*_], (*t*_*H* + *J*_, ∞ ), so that 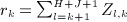. The full conditional distribution of *Z*_*k* + 1,*k*_, …,*Z*_*H* + *J* + 1,*k*_ given the probabilities *p*_1:*H* + *J* + 1_ is a multinomial distribution with sample size *r*_*k*_ and probability parameters *η*_*k* + 1,*k*_, …,*η*_*H* + *J* + 1,*k*_, where

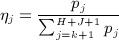
 for *j* = *k* + 1, …,*H* + *J* + 1.

#### 4.2.2. Sampling the probabilities *p*_1:*H* + *J* + 1_

Following the sampling of *Z*_*k* + 1,*k*_, …,*Z*_*H* + *J* + 1,*k*_, define *d* ′ _*k*_ to be the revised number of events in the interval (*t*_*k* − 1_,*t*_*k*_], which is the sum of the observed events and sampled events:

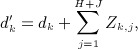
(27) where *d*_*k*_ and *Z*_*k*,*j*_ are the number of observed events and simulated events in the interval (*t*_*k* − 1_,*t*_*k*_], respectively. New probabilities *p*_1_,*p*_2_, …,*p*_*H* + *J* + 1_ are sampled from their full conditional distribution, which is another Dirichlet distribution:

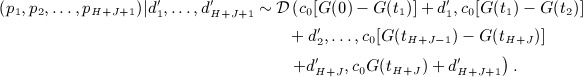


#### 4.2.3. Eliciting the prior for *θ* and calculating the assurance

To elicit the prior for the log hazard ratio *θ*, we only need to elicit the prior for the survival difference *ρ* at time *t*_0_ and then use simulation to obtain the prior for *θ* using (23). Simulated *S*_1_(*t*_0_) can be obtained when generating the survival rates using the Gibbs sampling approach. As before, the Monte Carlo simulation can be used to estimate (26). The R code to compute the assurance for the proportional hazards models is also available in the supporting software described in Section 3.2.

## 5. Numerical examples

In this section, we provide examples to illustrate how assurance is computed to inform the sample size choice under different model assumptions. We also present how elicited priors have an effect on the assurance. In each example, we suppose that a randomised controlled trial is going to be conducted to compare two treatment effects with an equal number of patients allocated to each treatment group. The sample sizes determined using power calculations are based on a 5*%* two-sided hypothesis test.

### 5.1. Exponential model

We first consider sample sizes based on power. We suppose that the trial has a 3-year recruitment period with a 2-year follow-up period and that 60*%* of patients receiving the standard treatment are expected to be alive after 5 years. For the power calculation, we consider an absolute 20*%* increase in patient survival for the experimental group.

Using Equation (9), we have the model parameters *λ*_1_ = 0.102 and *λ*_2_ = 0.0446. To achieve a specified power π*, the required sample size *N* is determined by solving

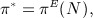
 where the power function *π*^*E*^ is given in Equation .

To calculate assurance *γ*^*E*^, an expert’s judgments about the 5-year survival rate in the control group *S*_1_(5) and the 5-year survival difference *ρ* are assessed using univariate elicitation methods. Suppose this yields *S*_1_(5) ∼ *B*(*a*_*s*_,*b*_*s*_) and *ρ* ∼ *N*(*m*_*ρ*_,*v*_*ρ*_). In the following, we look at three scenarios for the priors.

1. Scenario 1: *ρ* ∼ *N*(0.2,0.001) and *S*_1_(5) ∼ *B*(60,40).
2. Scenario 2: *ρ* ∼ *N*(0.2,0.05) and *S*_1_(5) ∼ *B*(60,40).
3. Scenario 3: *ρ* ∼ *N*(0.3,0.005) and *S*_1_(5) ∼ *B*(60,40).

In scenario 1, the prior *ρ* ∼ *N*(0.2,0.001) indicates a strong prior belief that the 5-year survival difference is around 0.2. In scenario 2, *v*_*ρ*_ = 0.05 implies that *P*(*S*_2_(5) − *S*_1_(5) > 0) = 0.769. In scenario 3, the prior *ρ* ∼ *N*(0.3,0.005) expresses the belief that *P*(*S*_2_(5) − *S*_1_(5) < 0) = 0.00003, that is, the experimenter believes that the experimental treatment has a very high probability of being superior.

**Figure 1:**
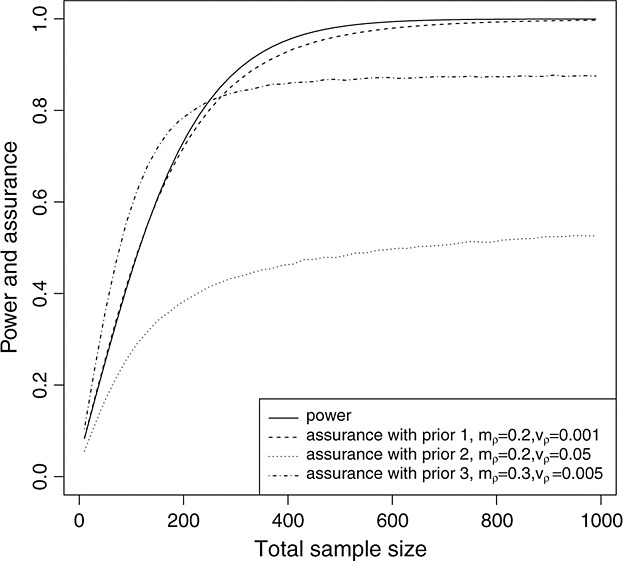
The comparison between power and assurance *γ*^*E*^ considering *ρ* ∼ *N*(*m*_*ρ*_,*v*_*ρ*_) and *S*_1_(5) ∼ *B*(60,40).

Figure 1 shows how the assurances differ given the different joint prior distributions. When an expert has strong beliefs (scenario 1) that the treatment effect will be close to that as specified in the power calculation, the required sample size informed by assurance is similar to that determined by the power calculation. In scenario 2, the assurance cannot exceed 80*%*, as the prior probability of the new treatment being superior is 76.9*%*. In scenario 3, a smaller sample size may be required to achieve an 80*%* probability of having a successful trial given the very high prior probability of the experimental treatment being superior.

### 5.2. Weibull model

We first consider determining sample sizes using the power function under the Weibull model. Suppose that the 1-year survival rate in the control group is expected to be 20*%* and to decrease to 10*%* at the end of the second year. We consider a two-sided hypothesis test of the null hypothesis of no change in the mean survival time between the control and experimental groups. For the power calculation, we suppose that the survival rate in the experimental group at 1 year is 30*%* and at 2 years is 20*%*.

Using Equations (17) and (18), the Weibull parameters in each group are *κ*_1_ = 0.52, *λ*_1_ = 1.61, *κ*_2_ = 0.42 and *λ*_2_ = 1.20. Using Equations (14) and (15), the mean and variance of the survival times in each group are *μ*_1_ = 0.75, 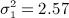, *μ*_2_ = 1.89, and 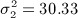. To achieve a specified power π*, the require sample size *N* is determined by solving

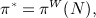
 where *π*^*W*^ is given in Equation (13).

To compute the assurance, we consider eliciting an expert’s opinion about the 1-year survival rate in the control group *S*_1_(1), the survival difference at 1 year between two groups, *δ*_12_, and the difference in survival probability between the experimental and control groups at 1 and 2 years, denoted by *δ*_11_ and *δ*_22_, respectively.

Suppose that we elicited quartiles for the uncertain quantities, as given in Table 1. Using the MATCH online elicitation tool, the distributions fitted to the elicited judgements are given later.


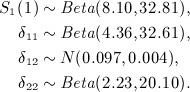


**Table 1:** Elicited quartiles of *S*_1_(1), *δ*_11_, *δ*_12_ and *δ*_22_.

	*S*_1_(1)	*δ*_11_	*δ*_12_	*δ*_22_
Lower quartile	0.15	0.08	0.05	0.05
Median	0.2	0.11	0.1	0.1
Upper quartile	0.23	0.15	0.14	0.12

**Figure 2:**
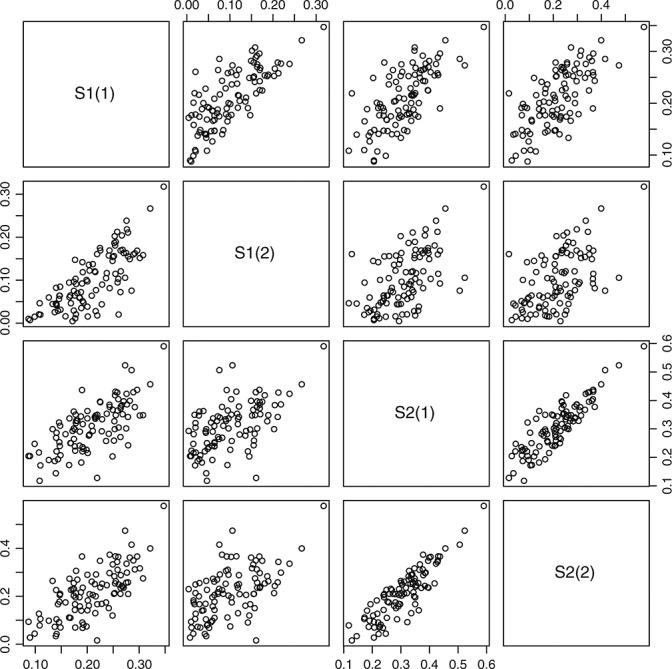
Scatterplots of *S*_*i*_(1) and *S*_*i*_(2) for *i* = 1,2, given the elicited quartiles.

**Figure 3:**
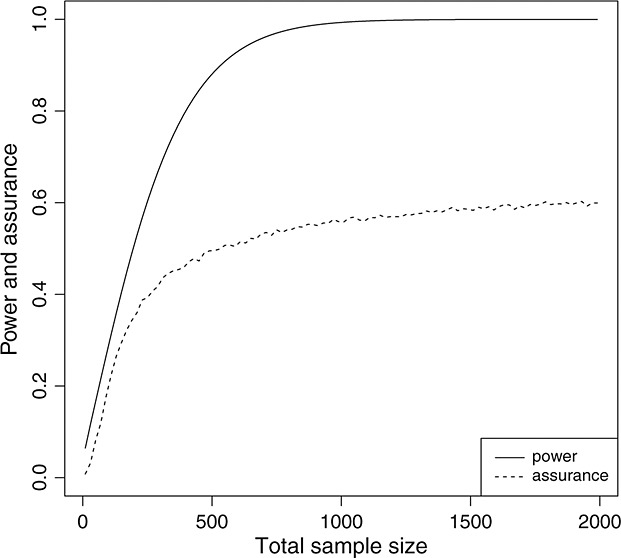
The comparison between power and assurance *γ*^*W*^ considering uncertainty in *S*_1_(1), *δ*_11_, *δ*_12_ and *δ*_22_.

Figure 2 shows how the four survival rates *S*_1_(1), *S*_1_(2), *S*_2_(1) and *S*_2_(2) are correlated given the elicited quartiles listed in Table 1. Figure 3 illustrates the comparison between the power and assurance functions. Given the quartiles of the uncertain quantities in Table 1, the prior probability that the experimental treatment is indeed superior is 73.9*%*, which cannot be exceeded by the assurance. The large difference in terms of power and assurance given a fixed large sample size is because uncertainty in the prior distributions has a large influence on the probability of success.

### 5.3. Proportional hazard model

Suppose that a trial is planned to have a 5-month recruitment period with a 5-month follow-up period. In our example, we use the data given in Kaplan and Meier 29 as the available information for the standard treatment. The data are 0.8, 1.0*, 2.7*, 3.1, 5.4, 7.0*, 9.2 and 12.1*, where ‘ * ’ denotes censored observations.

We first consider sample size calculations using the power function. For the power calculation, we consider an absolute 17.5*%* increase in the 7-month survival rate for the experimental treatment compared with the standard. Using the Kaplan–Meier estimate, the survival rate at 7 months for the standard treatment is *S*_1_(7) = 0.525, so the corresponding log hazard ratio *θ* is − 0.591. Using Equation (20), the probability, *P*_*e*_, that a patient will experience the outcome event during the trial is 0.42. To achieve a specified power π*, the required sample size *N* is determined by solving

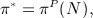
 where *π*^*P*^ is given in Equation (19).

Considering uncertainty in both the log hazard ratio and survivor function in the control group, the quantities that need to be elicited are the difference *ρ* in survival probabilities at 7 months between the experimental and standard treatment, and parameter function *α* of the Dirichlet process prior. Suppose we have already obtained the prior *ρ* ∼ *N*(0.175,0.01). Furthermore, an expert proposes the mean of the standard treatment survivor function to be an exponential distribution with a 5-month survival rate *S*_1_(5) of 50*%*. Hence, the parameter function *α* of the Dirichlet process is *α*([*t*, ∞ )) = *c*_0_ exp( − *λt*), where *λ* is given by

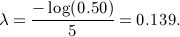


**Figure 4:**
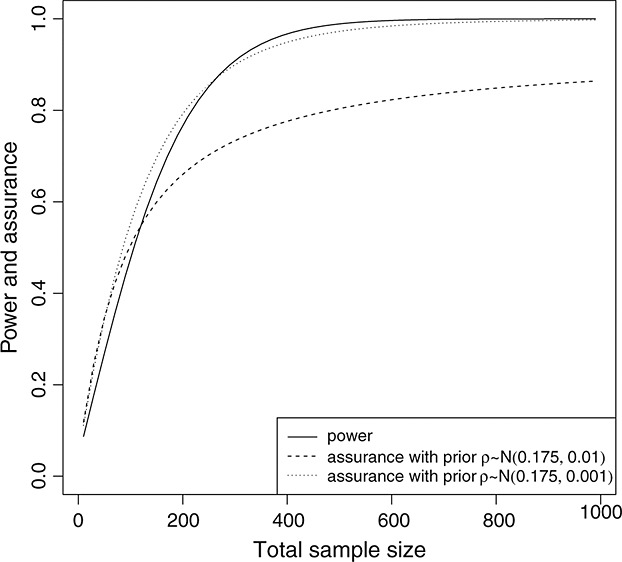
The comparison between power and assurance *γ*^*P*^ considering uncertainty in *θ* and *S*_1_(.).

We fix *c*_0_ at 1, which represents a fairly weak prior for the survival rates *S*_1_(.) with the data being dominant. Figure 4 shows the comparison between the power calculated on the basis of the Kaplan–Meier estimate of *S*_1_(.) and the assurance. The assurance (dashed line) in this case cannot exceed 95.5*%*, which is the prior probability of the experimental treatment being superior. If we had a stronger prior for *ρ*, for example, *ρ* ∼ *N*(0.175,0.001), then the calculated assurance would be very close to the power (as shown by the dotted line in Figure 4.

## 6. Summary

We have extended the assurance method to accommodate time-to-event outcomes in clinical trials, assuming one of three analysis methods, and we have made software available for implementing our methods. The reliability of an assurance probability will depend on the reliability of the elicited prior, and so it will be important to check the robustness of assurances to the choice of prior. However, the process of formally assessing the evidence in support of a new treatment and quantifying the attendant uncertainties could itself form a useful part of the trial planning process. Overall, we believe that it is clearly useful to know the probability of a trial producing a successful result, and in the context of clinical trial planning, the extra effort required in using the assurance method is relatively small.
